# Labial Angioedema and Bacterial and Viral Infection Following Hyaluronic Acid Injection

**DOI:** 10.7759/cureus.102791

**Published:** 2026-02-01

**Authors:** Macarena Olivares, Victor Mercado

**Affiliations:** 1 Dentistry, Instituto Chileno de Rejuvenecimiento y Optimización de Medicina Estética,, Santiago, CHL; 2 Otolaryngology - Head and Neck Surgery, Instituto de Neurorrehabilitación y Balance, Viña del Mar, CHL

**Keywords:** bacterial infection, hsv, hyaluronic acid injection, hyaluronidase, hypersensitivity, labial angioedema

## Abstract

Hyaluronic acid (HA) currently maintains a favorable safety profile in the field of aesthetic medicine. However, severe acute hypersensitivity reactions may occur, clinically defined as angioedema. This condition typically presents immediately after HA injection, with marked swelling, pain, and evident tissue distress secondary to edema and local vascular physiological alterations. These events are more frequently observed in the lips, and although reported cases are scarce, most involve this anatomical location.

The etiology of this uncommon complication is not fully understood. Proposed mechanisms include trace protein impurities within HA preparations, as well as the role of CD44-expressing mast cells, which exhibit strong adherence to tissue-bound HA. We present a case report of a patient who developed labial angioedema following HA injection, clinically associated with both viral and bacterial infection. The clinical presentation, diagnostic workup, therapeutic approach, and clinical outcome are described.

## Introduction

Hyaluronic acid (HA) is one of the most widely used fillers worldwide for aesthetic and reconstructive purposes. In 1999, the manufacturing process of non-animal-stabilized hyaluronic acid (NASHA) was modified to reduce bacterial protein content, which significantly decreased adverse event rates from 0.15% to 0.06%. The most relevant adverse effect was hypersensitivity, reported in approximately 1 out of every 5,000 treated patients [1]. These reactions were thought to be related not to the HA molecule itself, but rather to impurities derived from bacterial fermentation, residual proteins, bacterial fragments, or biofilm formation during manufacturing or injection processing [2].

Human mast cells express the HA-binding receptor CD44 and adhere directly to HA matrices. Through this interaction, HA may modulate mast cell activation and inflammatory responses, establishing a possible etiological link between HA implants and angioedema-type reactions [3]. Additionally, trauma to the different dermal layers during HA injection may induce local immunomodulatory alterations, triggering herpes simplex virus (HSV) reactivation in carrier patients, which can further exacerbate edema and tissue damage [4,5].

## Case presentation

A 29-year-old transgender patient presented to the emergency department with progressive lip swelling and intense pain a few hours after undergoing HA filler injection in the lips. The patient was initially treated on an emergency basis at a hospital, where she was prescribed an antibiotic regimen consisting of amoxicillin-clavulanic acid and oral prednisone. She did not respond to this treatment, and due to clinical worsening, she was referred to our clinic.

**Figure 1:**
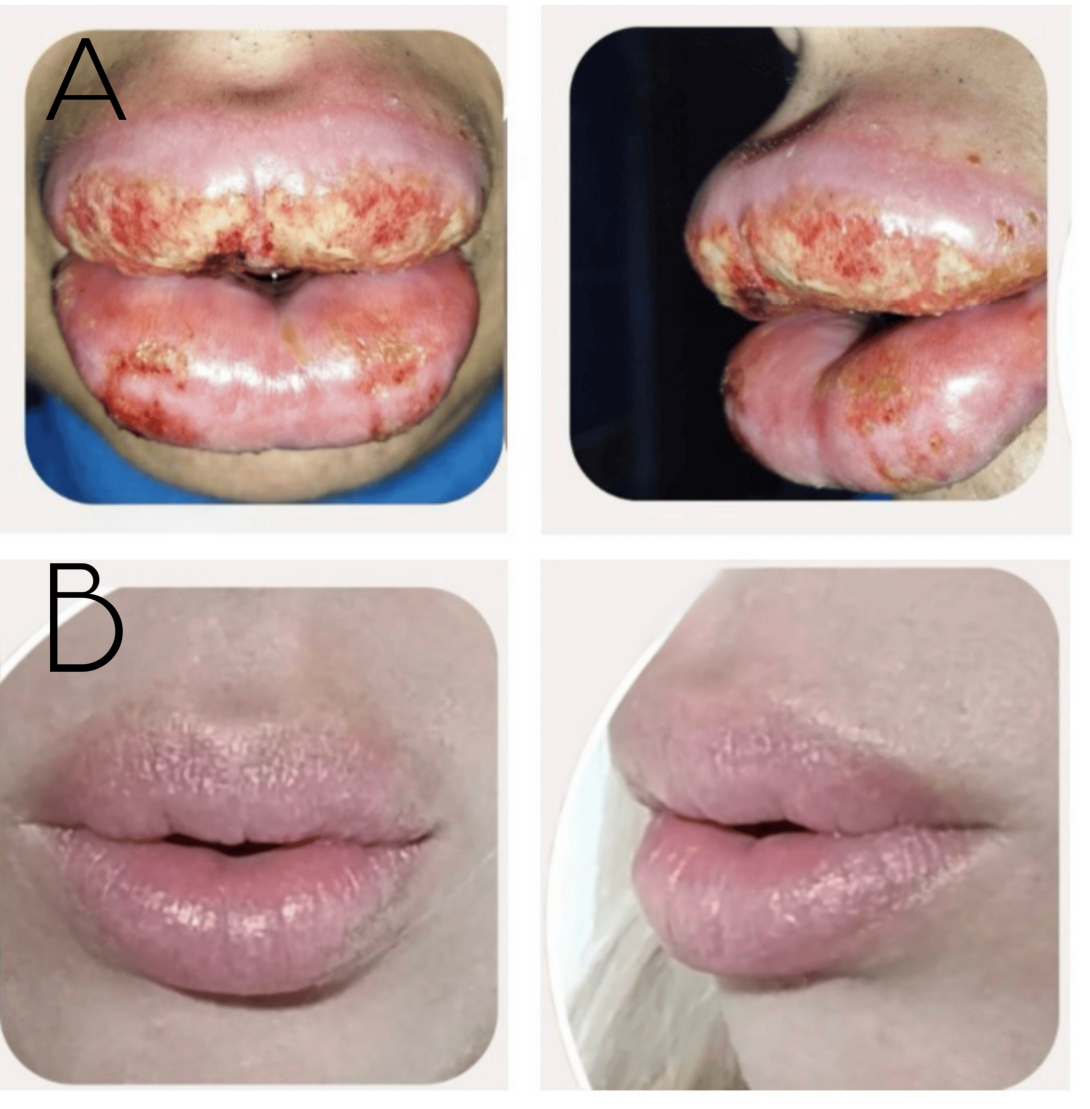
Sequential evolution from the initiation of treatment to its completion. (A) Initial presentation of the patient with angioedema and concomitant bacterial and viral infection. (B) Twenty-four days after the resolved case, with complete resolution of angioedema and no clinical evidence of bacterial or viral infection.

Within 48 hours, the clinical condition worsened, with the development of marked erythema, honey-colored crusts, grouped vesicles, increasing pain, and significant functional impairment affecting speech and oral intake. Upon specialized evaluation, the patient exhibited severe labial angioedema associated with bacterial superinfection and HSV reactivation (Figure 1A).

**Figure 2:**
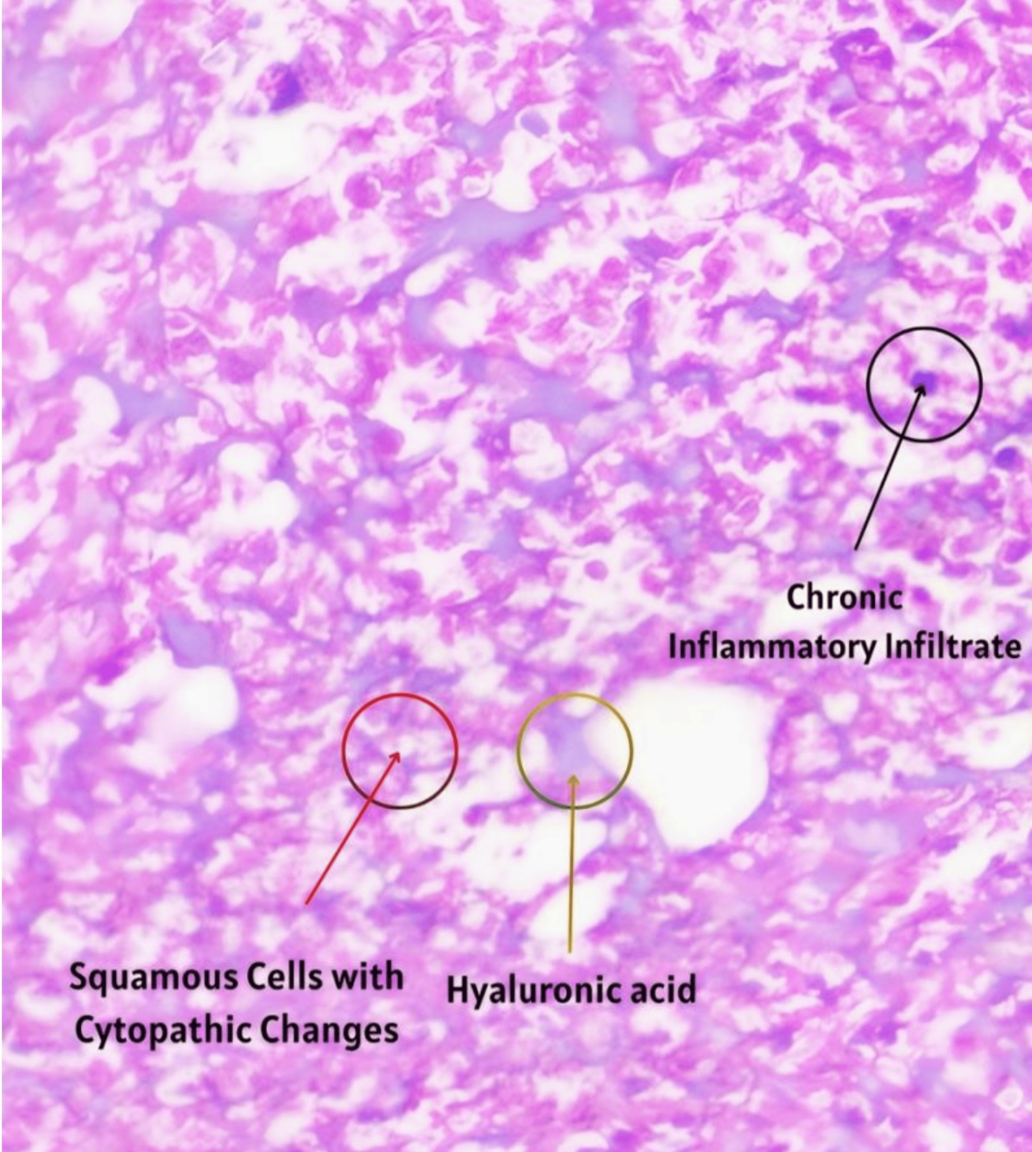
Compound optical microscope study (H&E staining technique) Keratin and squamous cells (red arrow) exhibiting cytopathic changes consistent with herpes simplex virus infection. Chronic inflammatory infiltrate with a lymphoplasmacytic pattern (black arrow) associated with a large amount of fibrin. Amorphous exogenous material showing grayish to slightly basophilic staining intensity, compatible with hyaluronic acid (yellow arrow).

With a compound optical microscope study (H&E staining technique), sections from an incisional biopsy of the upper lip show extensive areas of necrosis of the lining epithelium, with moist keratin and squamous cells exhibiting cytopathic changes consistent with HSV infection. An intense, diffuse chronic inflammatory infiltrate with a lymphoplasmacytic pattern is observed, associated with a large amount of fibrin. Focally, the presence of an amorphous exogenous material is identified, showing grayish to slightly basophilic staining intensity, compatible with HA. Histopathological diagnosis was nonspecific chronic inflammation with necrosis, cytopathic changes compatible with HSV infection, and exogenous material compatible with HA (Figure 2).

Systemic corticosteroids were discontinued, and a targeted treatment regimen was initiated, consisting of flucloxacillin 500 mg every six hours for seven days and acyclovir 400 mg every eight hours for 10 days. Once the acute infectious component was controlled, staged 2 hyaluronidase 1500 U sessions were performed to dissolve the injected HA, aiming to remove potentially contaminated material and reduce the risk of chronic biofilm-associated granulomatous reactions. Progressive clinical improvement was observed, with complete resolution of inflammation and regeneration of labial tissue without permanent sequelae (Figure 1B).

## Discussion

Angioedema following HA filler injection is a very rare but clinically significant complication. The reported incidence of hypersensitivity reactions to non-animal-stabilized HA ranges between 0.42% and 0.8%, depending on the product generation and impurity load [4]. These reactions may occur immediately or be delayed by weeks or months [4,5].

Mast cells play a central role in this process. Through CD44 receptors, mast cells bind directly to HA and, upon activation, release histamine, proteases, and proinflammatory cytokines that increase vascular permeability and produce the characteristic edema of angioedema [3].

In this patient, the additional presence of grouped vesicles and crusting clinically suggested HSV reactivation. Due to the severity of presentation and incomplete procedural details, a tissue biopsy was performed. Histopathological examination revealed keratinocyte involvement and cellular alterations of the labial epithelium. Similar histological findings have been reported in the literature [4], following HA injections in the lips complicated by HSV activation (Figure 2). Viral reactivation is facilitated by local trauma, inflammation, and transient immune dysregulation, all of which were present in this case [5,6]. Wang et al. concluded that herpes reactivation after HA injection is rare but requires heightened clinical awareness for accurate diagnosis, prevention, and treatment [7].

Bacterial infection following perioral HA injection has also been described in the literature. Studies suggest that multiple needle passes significantly increase the risk of filler contamination and subsequent infection [8].

The initial administration of systemic corticosteroids without excluding an infectious etiology likely exacerbated viral replication and bacterial proliferation, underscoring the importance of close observation and rigorous follow-up after HA filler procedures. Therefore, priority was given to treating both viral and bacterial infections, combined with enzymatic removal of the inflammatory biomaterial using hyaluronidase. This approach allowed resolution of the immunologic and infectious components of this severe reaction.

Flucloxacillin was selected due to its high activity against methicillin-sensitive Staphylococcus aureus, which remains the most frequently isolated pathogen in bacterial infections associated with HA dermal fillers, particularly in early and late infectious complications, as supported by the findings of Marusza et al. [9].

Beyond acute management, regenerative therapies such as low-level laser therapy and platelet-derived growth factors may support tissue recovery and reduce the risk of fibrosis following severe inflammatory injury [10].

## Conclusions

Angioedema is a very rare complication, particularly in the context of aesthetic procedures, and its occurrence in association with simultaneous viral and bacterial infection is exceptionally uncommon. This condition is characterized by a sudden and pronounced increase in tissue volume, often accompanied by pain, inflammation, and compromised local vascular physiology, which may rapidly lead to significant tissue distress. Early recognition of the clinical presentation is therefore crucial, as delayed diagnosis can result in progressive tissue ischemia, necrosis, secondary infection, and long-term functional or aesthetic sequelae. Prompt and accurate identification allows for the timely initiation of an effective, targeted therapeutic approach aimed at controlling inflammation, eradicating infection, preserving tissue viability, and minimizing the risk of permanent damage and adverse outcomes.
